# Development of a Novel DNA Aptamer Ligand Targeting to Primary Cultured Tumor Endothelial Cells by a Cell-Based SELEX Method

**DOI:** 10.1371/journal.pone.0050174

**Published:** 2012-12-04

**Authors:** Mst. Naznin Ara, Mamoru Hyodo, Noritaka Ohga, Kyoko Hida, Hideyoshi Harashima

**Affiliations:** 1 Laboratory of Innovative Nanomedicine, Faculty of Pharmaceutical Sciences, Hokkaido University, Sapporo, Hokkaido, Japan; 2 Division of Vascular Biology, Graduate School of Dental Medicine, Hokkaido University, Sapporo, Hokkaido, Japan; Wayne State University School of Medicine, United States of America

## Abstract

The present study used a spontaneous cell-based SELEX method (Systemic Evolution of Ligands by EXponential Enrichment) to produce DNA aptamers that specifically bind to cell surface proteins or biomarkers produced by primary cultured mouse tumor endothelial cells (mTECs). In solid tumors, new blood vessels are formed through an angiogenesis process, and this plays a critical role in cancer development as well as metastasis. To combat angiogenesis, an appropriate diagnosis and a molecular-level understanding of the different cancer types are now a high priority. The novel DNA aptamer AraHH001, developed in this study, binds specifically to mTECs with high affinity in the nano-molar range, but does not bind to normal skin endothelial cells (skin-ECs). The selected DNA aptamer was also found to bind to cultured human tumor endothelial cells (hTECs), isolated from a clinical patient with a renal carcinoma. The aptamer AraHH001 showed significant anti-angiogenesis activity by inhibiting tube formation by mTECs on matrigel. Interestingly, a confocal laser scanning microscopy examination of *in vitro* cellular uptake revealed that AraHH001 was assimilated by mTECs, and became co-localized in acidic compartments, as detected by labeling with Lysotracker Red. Therefore, the development of a specific DNA aptamer that binds to mTECs, as reported here for the first time, holds great promise not only as a therapeutic aptamer but also as a targeted molecular probe that appears to play a major role in angiogenesis, and for the development of a targeted new drug delivery system.

## Introduction

Angiogenesis-dependent tumor growth was first reported by Folkman in 1971 [1]. Preventing or inhibiting angiogenesis, which is associated with the increased vascularity necessary for tumor progression and metastasis, is a challenging issue in combating cancer. Tumor blood vessels provide nutrients and oxygen, and remove waste from tumor tissue, resulting in tumor progression. Tumor blood vessels differ from their normal counterparts, in that they are more permeable and, the thickness of the basement membrane is uneven. This suggests that tumor endothelial cells may express surface markers that are different from those found on normal cells. Tumor blood vessels contain tumor endothelial cells that might be genetically normal and stable, even though these endothelial cells are structurally and functionally abnormal. Since progressive tumor growth and metastasis depend on angiogenesis, inhibiting angiogenesis by targeting tumor endothelial cells represents a promising strategy for cancer treatment [2], [3].

Our rationale for targeting tumor endothelial cells in our current project is based on the following assumptions: A single tumor endothelial cell can support many tumor cells. Thus, targeting endothelial cells might be a much more effective strategy than targeting the actual tumor cells themselves, since the tumor endothelial cells from all tumor types are very similar. Thus, the development of an ideal anti-angiogenic drug might be useful for the treatment of a wide variety of cancers. Tumor endothelial cells are considered to be genetically stable, and, thus might not acquire drug resistance, unlike tumor cells. Although recent studies have suggested that tumor endothelial cells might be different from normal endothelial cells, they might also be heterogeneous between organs or tumor types [4]–[6].

The *in vitro* SELEX method was used to develop aptamers. The SELEX method for selecting an aptamer from combinatorial libraries by an iterative *in vitro* selection procedure was first reported independently by two research groups, Ellington’s and Tuerk’s in the early 1990s [7], [8]. We developed a new DNA aptamer using the method known as Cell-SELEX, a modification of SELEX against complex live target cells [9]–[15]. Aptamers, ssDNA, ssRNA or peptide molecules [16], are very easy to produce, are generally nontoxic, and have low molecular weights (8–15 kDa) [17], and their target binding is very specific and selective. Aptamers can be chemically modified to enhance their stability in biological fluids, and because of their small size, they can easily and rapidly diffuse into tissues and organs, thus permitting faster targeting in drug delivery. Aptamers are a new class of molecular probes and are comparable to antibodies, in terms of their specificity and affinity (µM to pM range),with the potential for use in conjunction with a wide range of target molecules, including small molecules such as dyes, metal ions, amino acids and nucleotides, biomolecules such as nucleic acids and proteins, molecular complexes, viruses, whole organisms, or even live cells. Since aptamers can be produced with well defined secondary and three dimensional structures, they can bind with high affinity to their targets [18]–[26]. Extensive research on aptamers has shown that they have great potential for use in a variety of areas, including diagnosis, therapy, biomarker identification, ligand targeting for the delivery of molecules, *in vivo* imaging, and biosensors. These advantages, as well as recent applications of aptamers in different biomedical areas and their potential uses, have increased their priority for development [19]–[31].

**Figure 1:**
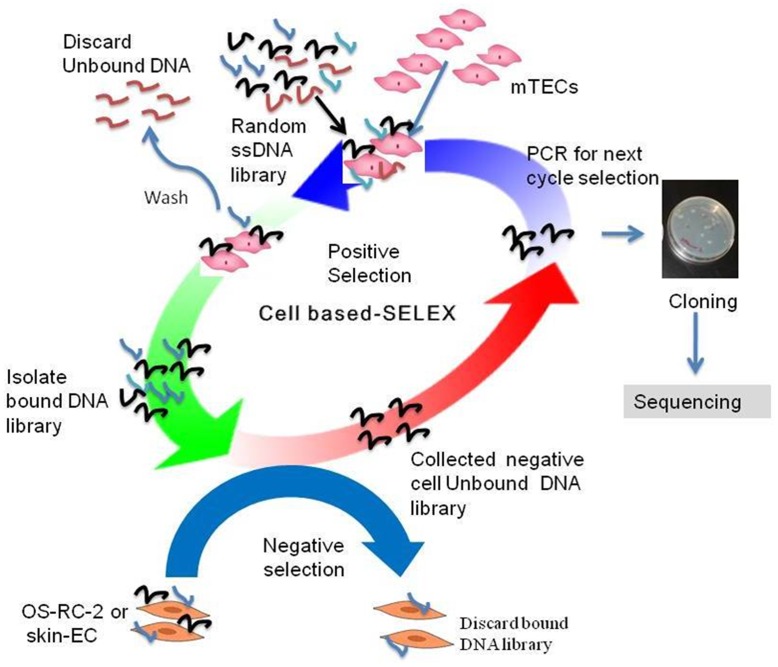
A schematic representation of the cell-based SELEX method used for the selection of DNA aptamer. In short, a 200 pmol ssDNAs library was incubated with mTECs on ice for 45 minutes. A five molar excess BSA and yeast tRNA was used to reduce nonspecific binding. After washing bound ssDNA from cells were eluted by heating at 95°C for 5 minutes. Selected ssDNA pools were subjected to amplify with fluorescent tag to start the next cycle. At cycles 11 and 12, negative selection was done using skin-ECs and OS-RC-2 cell lines along with the positive selection. After a successful 12 cycle’s selection, the enriched pool of ssDNA was subjected to clone and sequence for the identification of the individual aptamer.

We successfully developed a DNA aptamer that binds specifically to target cells, mTECs. The findings reported here show that the mTEC specific DNA aptamer might easily differentiate between healthy cells and diseased cells at each molecular level. This differentiation is clearly an added advantage, and could contribute significantly to our understanding of diseases with the mechanistic aspects associated with their development. This difference is a potentially useful tool in diagnosing disease and designing a targeted drug delivery system [32], as well as in stimulating research regarding target biomarker identification and purification.

**Figure 2:**
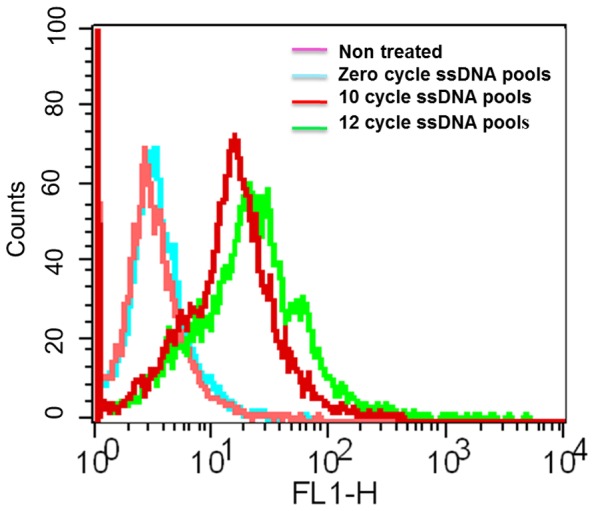
Flow cytometry binding assay of selected FITC labeled ssDNA pools with mTECs. Enrichment in binding random ssDNAs in the selection was observed by flow cytometry analysis. Here the curve represents non-treated cells and treated cells with a zero cycle ssDNA 10 and 12 cycle ssDNA pools respectively. 400 pmol of ssDNA library was used in each case. A zero-cycle ssDNA pool was used to observe the differentiation of enrichment from the starting point of selection with increasing cycle of selection.

## Materials and Methods

### Synthesis and Purification of a DNA Library

The random ssDNA library used for cell-based selection was synthesized by the solid phase phosphoramidite method [33]. The synthesized ssDNA library was purified, and the DMTr group was de-protected with 80% acetic acid and then was further purified by HPLC followed by quantification by UV-visible spectrophotometry. The purified 82 mer ssDNA library was confirmed by 3.8% agarose gel electrophoresis. The sequence of this library was comprised of 40 central randomized sequences flanked by 21 nucleotide forward primers and 21 nucleotide reverse primer shown below at 5′ and 3′ end respectively.

**Table 1:** List of DNA aptamers with insert sequences.

Aptamer	Truncated version of insert aptamer sequences
AraHH001	ACGTACCGACTTCGTATGCCAACAGCCCTTTATCCACCTC
AraHH004	GTTGTAGTATGGTGGTGCGGTGCAGGTGGGAAT
AraHH008	GTGTATGTGGCTATAGTACGCGATGTTCGT
AraHH009	CGGGTATGGGGTGGTGCTATGTGTATATG
AraHH022	GTGTTACGTGACCGAGACGGTTAGTTCTATGGTCAAGG

5′-CGTAGAATTCATGAGGACGTT -N40- AGCTAAGCTTACCAGTGCGAT 3′.

### Selection Buffer

We used the 1X selection buffer in all experiments. The 1X selection buffer was prepared by dissolving 50 mM Tris-HCl (pH 7.5), 5 mM KCl, 100 mM NaCl, 1 mM MgCl_2_, 250 mM sucrose and 0.1% sodium azide. A five molar excess yeast tRNA and BSA was used as to prevent non-specific binding. The selection buffer was sterilized with Millipore Steriflip (Millipore Corporation, Billerica, USA) before use.

**Figure 3:**
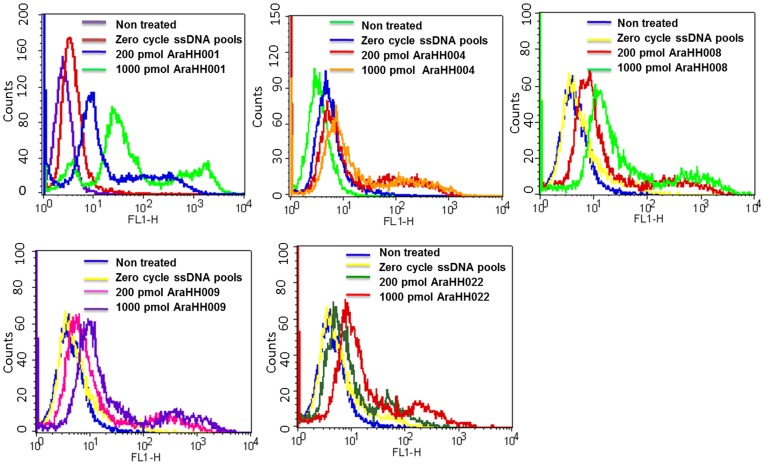
Identification of selected FITC labeled DNA aptamers on mTECs by a flow cytometry assay. Flow cytometry data represents binding assay of five FITC-labeled DNA aptamers with mTECs. In each DNA aptamer binding assay has shown the result of non treated mTECs, treatment mTECs with 200 pmol FITC labeled zero cycle ssDNA pools, treatment mTECs with 200 pmol DNA aptamer and 1000 pmol DNA aptamer independently.

### Cells and Cell Lines

We used a series of primary cultured cells such as normal skin endothelial cells (skin-ECs) as a negative control, which was isolated from normal mice skin. Mouse tumor endothelial cells (mTECs) as target for selection, was isolated from human tumor xenografts of melanoma tumor cell (A375SM) into nude mice. A375SM, a super-metastatic human malignant melanoma cell, was kindly gifted by Dr. Isaiah J. Fidler, (University of Texas, M.D. Anderson Cancer Centre, Houston). Other tumor endothelial cells included mOS-RC-ECs, was isolated from human tumor xenografts of OS-RC-2 tumor cells (renal tumor) into nude mice. OS-RC-2, [34] a human renal clear carcinoma cell was purchased from the RIKEN Cell Bank (Tsukuba, Japan). We have used human tumor endothelial cells (hTECs) was isolated from renal carcinoma patients excised tumor kidney tissues. The cell lines used included human umbilical vein endothelial cells (HUVECs), which was purchased from Clonetics. Human Dermal Microvascular Endothelial cells (HMVECs) were purchased from Lonza, Walkersville, MD, USA. Human Renal Plasmid incorporated a super-metastatic human malignant melanoma cells (RFP-SM), was generous gift from Dr. Isaiah J. Fidler, of the M.D. Anderson Cancer Centre, Houston, TX.

**Figure 4:**
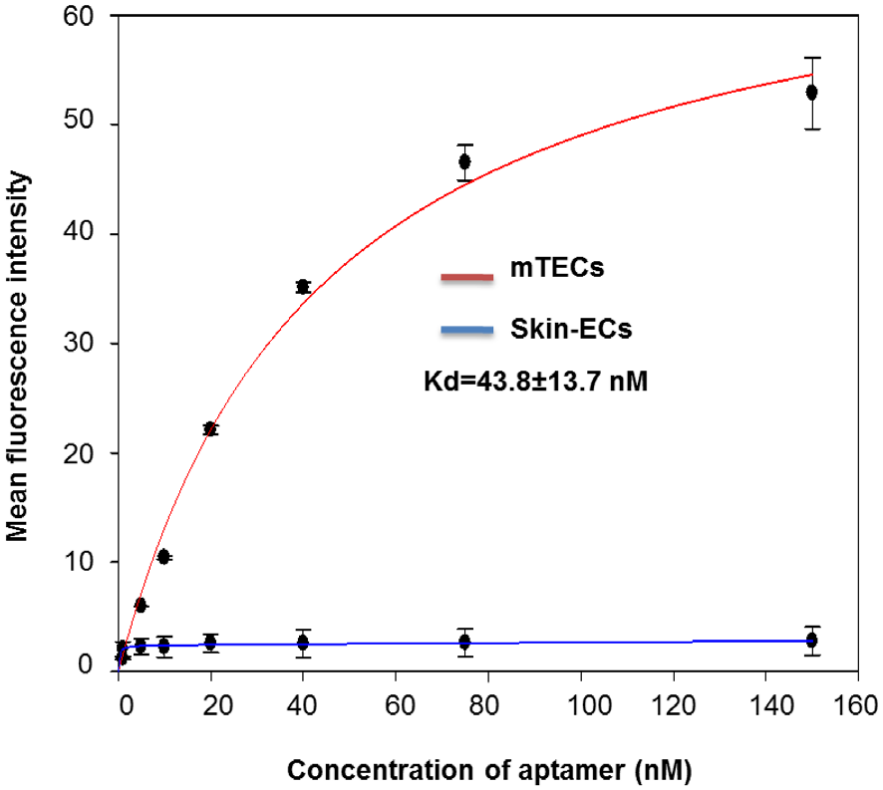
Determination of binding affinity, Kd value of the DNA aptamer AraHH001 by a flow cytometry. The Binding affinity of AraHH001 was determined by a flow cytometry using FITC-AraHH001 to mTECs and to skin-ECs. The average mean fluorescence intensity of varying concentration of FITC-AraHH001obtained was plotted to determine dissociation constant Kd. The experiment was repeated three times and a Error bar represents the standard deviation of means.

### Isolation of Skin-ECs, mTECs, mOS-RC-ECs, and hTECs

All experiments involving animals and their care were carried out following Hokkaido University guidelines, and the protocols were approved by the Institutional Animal Care and Use Committee. Human tissue samples were obtained from excised renal cell carcinoma patients at Hokkaido University Hospital, Hokkaido, Japan. Informed consent was obtained from all patients before samples were used. The protocols were approved by the Institutional Ethics Committee of Hokkaido University, and written informed consent was obtained from each patient before surgery. Endothelial cells were isolated as previously described [4]–[6], [41]–[43]. Briefly, normal endothelial cells (skin-ECs) and mTECs and mOS-RC-ECs were isolated using magnetic cell sorting system (Miltenyi Biotec, Tokyo) with CD31 antibody. CD31-positive cells were sorted and plated on to 1.5% gelatin-coated culture plates and grown in EGM-2 MV (Clonetics, Walkers, MD) and 15% FBS. Diphtheria toxin (DT) (500 ng/mL; Calbiochem, San Diego, CA) was added to mTECs subcultures to kill any remaining human tumor cells and to skin-ECs subcultures for technical consistency. However, DT does not interact with mouse HB-EGF and murine ECs survive this treatment. The isolated skin-ECs were purified by a second round of purification using FITC-BS1-B4 Lectin and anti-FITC beads (Miltenyi Biotec). Excised human renal carcinoma tissue was processed by a magnetic bead cell sorting system using the IMag cell separating system (BD Bioscience) to promptly isolate hTECs as presented above with mouse anti-human CD31 antibody (BD Pharmingen). hTECs were isolated by the IMag cell separation system according to the instruction of the manufacturer using anti mouse IgG1 Magnetic Particles (BD Biosciences). They were plated cultured by in EGM (Lonza) and 15% FBS.

**Figure 5:**
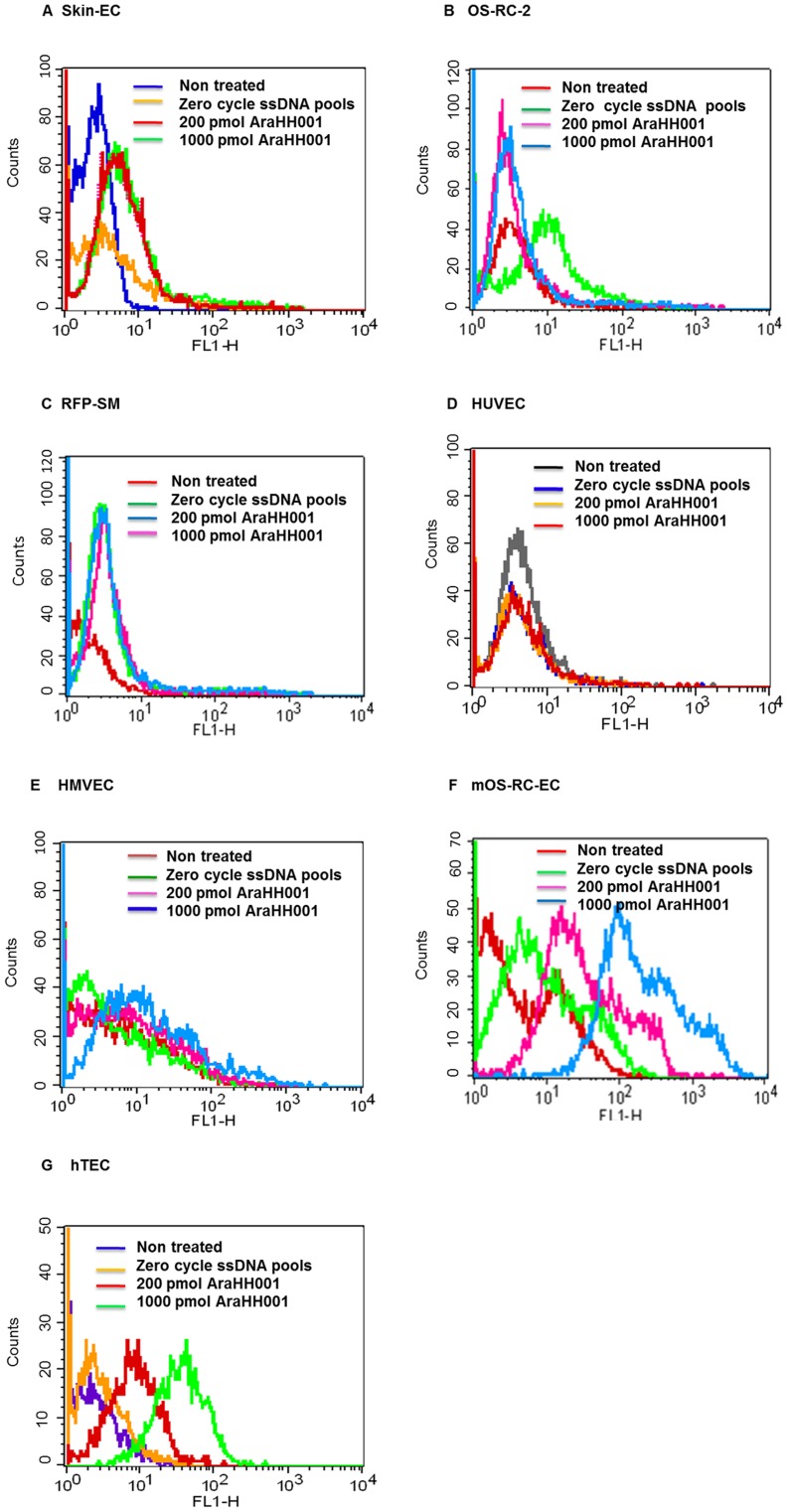
Binding assay of selected FITC-labeled DNA aptamer AraHH001 against a series of cells, and cell lines by a flow cytometry assay. A flow cytometry binding assay of AraHH001 with A. Normal skin-ECs, B. OS-RC-2, C. RFP-SM, D. HUVEC, E. HMVEC, F. m OS-RC-EC, and G. hTEC. In all cases, the results show non-treated, treated with 200 pmol FITC labeled zero cycle ssDNA pools, treated with 200 pmol and 1000 pmol FITC-labeled DNA aptamer AraHH001.

### Maintenance of Cell Cultures

mTECs, mOS-RC-ECs, hTECs, Skin-ECs, and HMVECs were cultured using a special medium, called Endothelial growth medium-2 (EGM-2 MV). For culturing OS-RC-2 cell lines we used RPMI-1640 medium with 10% fetal bovine serum (FBS). HUVECs, and RFP-SM cell lines were cultured in EBM-2 medium with 2% FBS, and minimum essential medium (Gibco, Grand Island, NY) supplemented with 10% FBS respectively. To prevent microbial growth we used penicillin 100 unit/mL and 100 µg/mL streptomycin in all mediums. Cell cultures were maintained at 37°C in a 5% CO_2_ incubator with 95% humidity. For regular cell cultures we used 0.1% trypsin to dissociate the cells from the surface of the culture dish. However, during the selection and flow cytometry analysis, we used special cell culture dish named RepCell [35].

**Figure 6:**
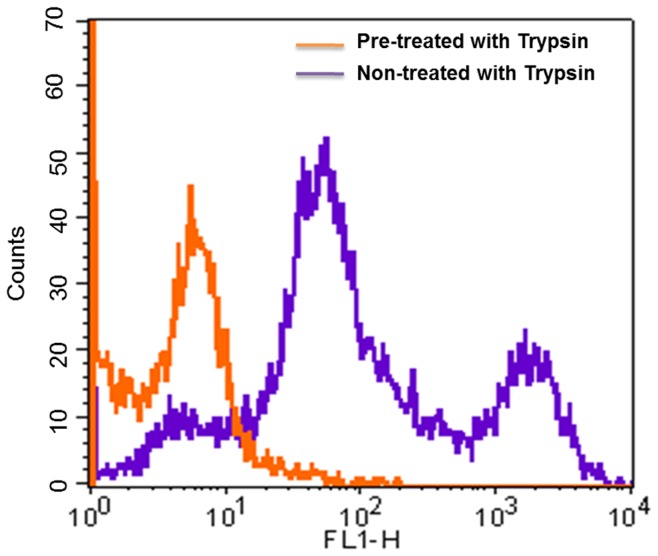
Binding assay of selected FITC-labeled DNA aptamer AraHH001 to trypsin pre-treated mTECs and trypsin non-treated mTECs. 1000 pmol of the AraHH001 aptamer was used in a binding assay using trypsin pre-treated and non-treated cells. The orange bar shows that the AraHH001 aptamer does not bind to trypsin pre-treated mTECs, while the Purple bar shows a high binding of the AraHH001 to trypsin non-treated mTECs.

### Generation, Extraction and Purification of ssDNA Library

The Asymmetric PCR method was used for generating the ssDNA library, as originally conceived and used by Gyllensten & Erlich [36]. For the preparation of ssDNA, asymmetric PCR was performed with two sequential PCRs. The 1st PCR was performed with 50 fmol of templates dsDNA per reaction. This PCR reaction was performed with a volume of 50 µL with 20 pmol of forward primer (5′-CGTAGAATTCATGAGGACGTT), 20 pmol of reverse primer (AGCTAAGCTTACCAGTGCGAT-3′) using five units of Taq DNA Polymerase (Qiagen). The PCR reaction was applied with 95°C for five minutes as the initial de-naturation, followed by 10 cycles of standard PCR protocols, by denaturing steps at 95°C for 30 seconds, an annealing step at 50°C for 30 seconds, and an elongation step at 72°C for 60 seconds. A final extension step was performed at 72°C for four minutes. The 2nd PCR was performed directly with 5 µL of 1st PCR product. This PCR reaction used a volume of 50 µL with only 50 pmol of forward primer (5′-CGTAGAATTCATGAGGACGTT), and no reverse primer using 5 units of Taq DNA polymerase (Qiagen). The PCR reaction was applied at 95°C for 5 minutes as initial denaturation, followed by 25 cycles’ standard PCR protocols, with a denaturing step at 95°C for 30 seconds, an annealing step at 50°C for 30 seconds and an elongation step at 72°C for 60 seconds. A final extension step was performed at 72°C for 4 minutes. The PCR product was run on 20% native PAGE gel with 1X TBE buffer, the gel was stained with ethidium bromide, and the desired band was cut with a thin spatula under a UV-trans-illuminator (Kurabo, Japan). The cut gel particles were crushed into very small particles, and the ssDNA eluted with 1X PBS in 37°C with shaking for 1 h, 2 h, 4 h and overnight by changing the solvent. The extracted ssDNA was purified, and desalted by illustra™ NAP-5 column chromatography (GE Healthcare, UK), and the purity became check on 20% native PAGE.

**Figure 7:**
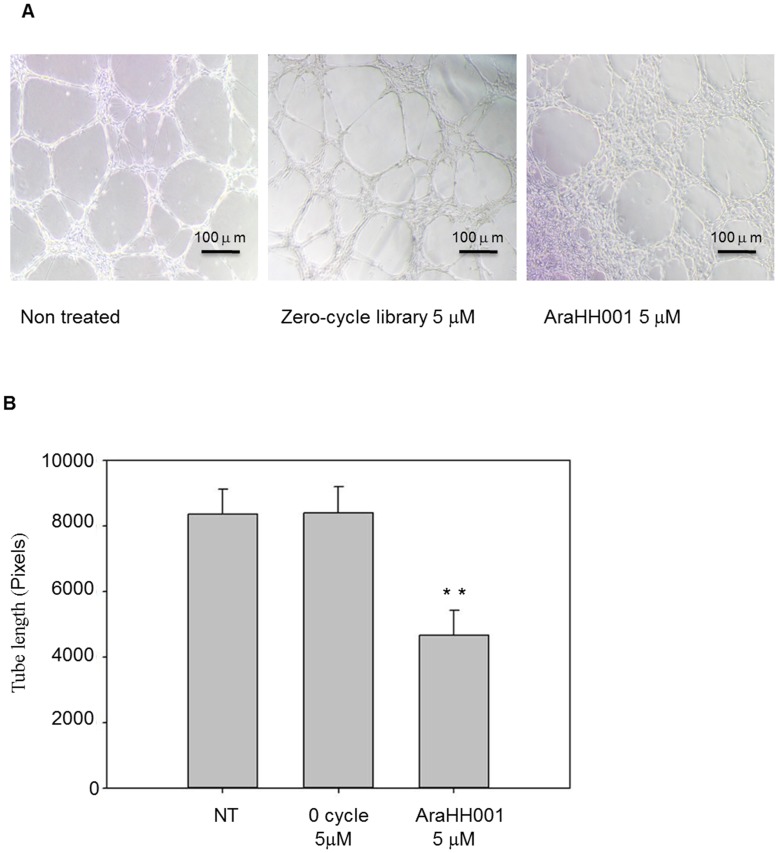
*In vitro* tube formation assays to measure anti-angiogenesis activity of the AraHH001. The tube formation was observed after 20 hours incubation with non-treated, zero-cycle library treated as a negative control, and AraHH001 treated mTECs on matrigel. **A.** Microscopic observation of tube formation of mTECs on matrigel. The tube formation was prohibited by AraHH001 treated mTECs on matrigel. Scale bar 100 µm. **B.** Quantitative analysis of tube formation. The tube length was calculated in pixels using one-way ANOVA after SNK test in each sample, and performed statistical analysis**.** Data are represented as mean± SD (n = 3 in each cases). **P<0.01 show significant differences NT and zero-cycle vs. AraHH001 treated group.

### A Cell-based SELEX Method for the Selection of DNA Aptamer

To start a cell-based selection, 200 pmol of 82 mer random ssDNA library with fluorescein isothiocyanate (FITC) tagged at the 5′ end and a biotin tag at the 3′ end was used. The ssDNA library was first dissolved in 1X selection buffer (500 µL), and heated in a thermo-block at 80°C for 10 minutes, and then cooled slowly to form a secondary structure necessary for binding with the target cell surface protein. A five molar excess of yeast tRNA and BSA was added to prevent nonspecific binding. For better selection, the procedure was modified to avoid the use of trypsin so that not to destroy cell surface proteins or biomarkers to which aptamers could be bound. Instead of trypsin we used temperature responsive dishes to detach adhesive cells, called RepCells (Cell Seed Inc., Japan), a polymer is fixed to the surface of the culture dish at a nano-thickness level. The surface then becomes slightly hydrophobic at temperatures above 32°C, thus allowing adhesion of the seeded cells. RepCells surfaces become super hydrophilic below 32°C, allowing spontaneous cell surface detachment. In considering cost effectiveness, normal culture conditions of cells were followed using trypsin in normal 10 cm dishes. Two to three days before of doing experiments we always transferred the cells from normal 10 cm dishes to 10 cm RepCells dishes. The cells were collected by centrifugation (1000×g, 4°C, 10 mins), and washed three times with 1X selection buffer and filtered with 40-µm cell strainer (BD Falcon) to remove clumped cells. The ssDNA libraries were then incubated with 1×10^6^ of our target mTECs on ice for 45 minutes. After incubation, the cells were spun down (5000×g, 4°C, 5 mins) to remove supernatant that contains unbound libraries. The cells-ssDNA complexes were then washed three times with 1X selection buffer to remove completely unbound DNA libraries. Finally, the bound DNA libraries were eluted by heating at 95°C for 5 minutes.

**Figure 8:**
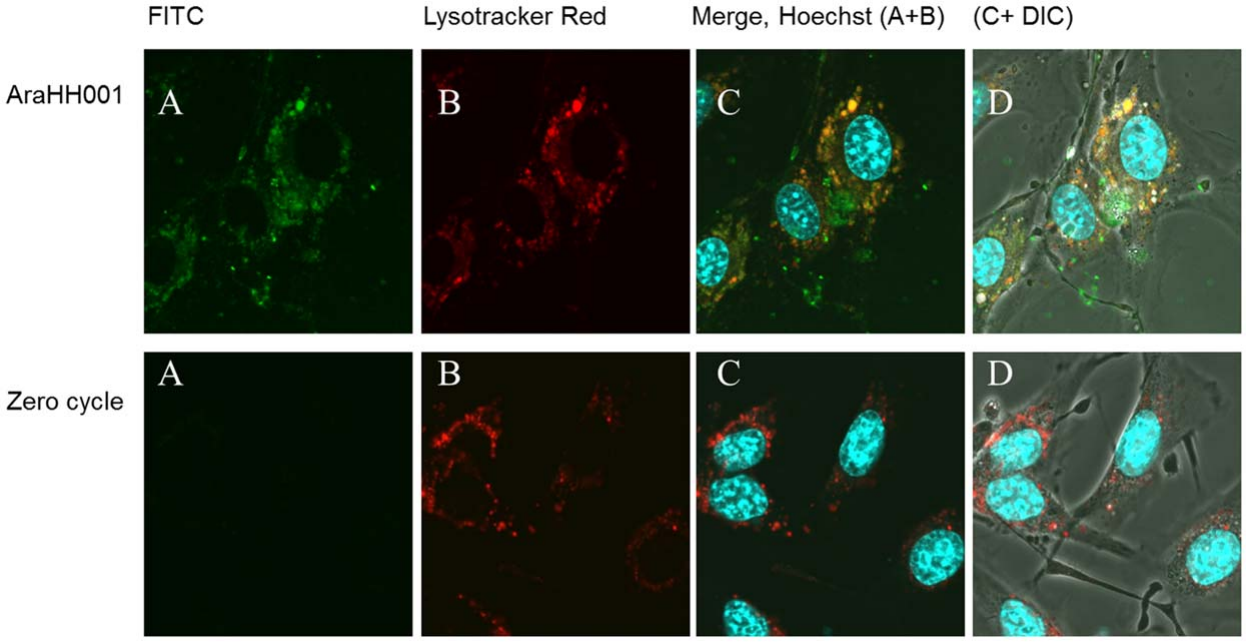
*In vitro* cellular uptake of the AraHH001 aptamer and control zero cycle pools in acidic compartments of mTECs. mTECs was incubated with FITC-tagged aptamer AraHH001 and zero cycle pools at 37°C for 30 minutes to check internalization, and were stained live nuclei with Hoechst 33342 and acidic compartments with LysoTracker Red and analyses with confocal laser scanning microscopy. **A.** (FITC-tagged aptamer AraHH001 and zero cycle pools). **B. (**Uptake of Lysotracker red into the acidic compartments of mTECs). **C.** (merge of images, Hoechst, A, and B). **D.** (merge of image Hoechst, A and B+DIC).

The bound DNA was purified by phenol-chloroform extraction followed by ethanol precipitation. For the phenol chloroform extraction, we added 500 µL of phenol-chloroform-isoamyl alcohols to 500 µL of bound DNA. After mixing well, the sample was centrifuged at 15000×g for 5 minutes, and the supernatant collected as the product. This procedure was repeated three times. We performed the same extraction three times with fresh chloroform.

For ethanol precipitation, 3 M sodium acetate (50 µL) was added along with 1200 µL of chilled ethanol to 500 µL of the selected DNA library, followed by incubating overnight at –80°C. The next day, the sample was subjected to spin down at 15000×g for 1 h, remove the supernatant and dry up the precipitate to dissolve with 10 mM Tris-HCl.

To start the next selection cycle, we optimized the PCR conditions and amplified the ssDNA library by asymmetric PCR. We performed total of 12 rounds of selections. After 10 rounds of selections, we performed two rounds of negative selection along with a positive selection. For the negative selection, we used skin-ECs and OS-RC-2.

### Aptamer Binding Assay by Fluorescence Activated Cell Sorting Analysis (FACS Analysis)

To estimate the extent of enrichment of binding of the ssDNA libraries as well as to produce a high affinity DNA aptamer, the first selected libraries in 500 µL of selection buffer, were heated at 80°C for 10 minutes, and cooled slowly to permit secondary structures to be formed, which are necessary for binding to the target cell surface protein. We added a five molar excess of yeast tRNA and BSA as a nonspecific binding agent. It should be noted here, that we were prepared cells using RepCell dishes to avoid damage to cell surface proteins caused by using trypsin, and the cells were filtered using a 40-µM Cell Strainer (BD Falcon) to remove clumped cells. 1×10^6^ cells were incubated with the selected library at different cycles during the selection period, and with the control zero-cycle ssDNA library on ice for 45 minutes respectively. The cells were spun down (5000×g, five minutes 4°C) to remove the supernatant that contains unbound DNA. The cells were washed three times with 1X selection buffer. Finally, we performed a fluorescence analysis of the sample on a FACS Calibur flow cytometer (BD Biosciences, San Jose, CA, USA) by counting 10000 events.

The selected aptamer binding affinity was measured by incubating 5×10^5^ mTECs with varying concentrations (1 nM-150 nM) of the selected aptamer pool in 500 µL of selection buffer containing 5 molar excess of BSA on ice for 50 minutes in the dark. The cells were washed two times with 1X selection buffer followed by flow cytometry measurements by 10000 counts. The mean fluorescence intensity of the target cells bound to the aptamer was used to calculate the specific binding. The equilibrium dissociation constant Kd was measured by fitting the dependence of the fluorescence intensity of specific binding on the concentration of the ligands to the equation *Y = B*max *X*/(*K*d+*X*) using the SigmaPlot 12 (Systat Software Inc., USA).

### Cloning and Sequencing of 12 Cycles Selected Library

Another important step was required to complete the successful selection, e.g., the selected libraries need to be cloned, and analyzed to confirm that the compound was, in fact, a high affinity DNA aptamer that was specific for mTECs. For the cloning experiment, we were using a TOPO TA cloning kit (Invitrogen). To determine the individual sequence for the selected colonies we performed sequencing experiments based on the Sanger method by using a Big Dye Kit. An ABI prism 3130-AVANT Genetic analyzer was used to complete the sequencing analysis.

### Trypsin Treatment on mTECs

mTECs were pretreated with 0.05% trypsin-EDTA solution for 10 minutes at 37°C. Cells were washed with 1X selection buffer, and incubated the cells with the AraHH001 for the binding assay, already detailed in aptamer binding assay by FACS in the experimental section.

### Tube Formation Assay

The tube formation experiment was carried out as described previously [37]–[39]. Briefly, 500 µL Matrigel was transferred (BD Biosciences, San Josa, Ca, USA) to each well of a 24-well dish plates and incubate 37°C 30 minutes to solidify matrix solution. mTECs was harvested, and re-suspended in EBM-2 with 0.5% FBS, and seeded at a density 1X 10^5^ cells per well followed to incubate with 5 µM aptamer AraHH001, and 5 µM zero-cycle library as a negative control at 37°C for 20 hrs. Tube formation was observed with an inverted microscope. Tube length was measured of random fields with ImageJ software.

### Confocal Laser Scanning Microscopy (CLSM) for *in vitro* Cellular Uptake Study

An internalization study with the AraHH001 was performed by confocal microscopy at 37°C [40]. We prepared 3.5 cm glass-bottom dish (1×10^5^ mTECs/2 mL medium), and incubated overnight at 37°C. On the next day, the medium was removed, and washed the preparation by adding 2 ml PBS followed by incubation with 500 pmol FITC-tagged AraHH001 for 30 minutes. The nuclei were then stained with Hoechst (1 mg/mL), the acidic compartment with LysoTracker Red, 0.5 µM for 15 minutes before finishing the incubation. After washing with 1X PBS buffered solution two to three times, added 1 mL of Kreb’s buffer and observed under CLSM. The excitation/emission range of FITC, Hoechst and Lysotracker Red were 494/518 nm, 350/461 nm, and 577/590 nm respectively.

## Results

### Selection of DNA Aptamers with the Cell-SELEX Method

We applied the cell-based SELEX method [10]–[15] to generate high affinity DNA aptamers, as shown in detail (Fig. 1). The focus of our current project involves the use of isolated primary cultured mTECs as the positive target, and primary cultured normal skin-ECs [4]–[6] and OS-RC-2 cell lines for negative selection. We started the 1^st^ cycle selection with 200 pmol random 40 mer ssDNA libraries flanked with a 21 mer primer. We performed 12 cycles to produce enriched high affinity random 40 mer ssDNA pools in sufficient quantities to finish the selection. After 10 cycles, we applied negative selection along with positive selection, because negative selection sometimes hinders positive selection. We checked the enrichment of the entire 12^th^ round selections by flow cytometry. The enrichment of the selected ssDNA pools was proportional to the increase in cycle number. At the 10^th^ and 12^th^ round selection, the affinity of the enriched random ssDNA pool was substantial, as evidenced by flow cytometry (Fig. 2). The binding affinity of the ssDNA pools to mTECs was clearly enhanced with an increase in the number of selection cycles, thus proving that the ssDNA libraries with higher binding affinity to the target cells were enriched**.** We performed negative selection at the 11^th^ and 12^th^ cycles along with positive selection, to remove the DNA libraries that could bind to the skin-ECs or OS-RC-2 cell lines. To obtain DNA aptamers that were selective for mTECs, negative selections were also important. The enrichment of the library was sufficiently high after the 12^th^ round of selection to produce a library for cloning and sequencing to obtain individual DNA aptamers. The entire selection procedure was modified to avoid the use of trypsin. Instead of trypsin, we used RepCells (CellSeed Inc., Japan), which are temperature-responsive dishes.

### Identification of DNA Aptamers

After 12 rounds of selection, the random ssDNA pools that bound to mTECs with high affinity were used in cloning experiments, using a TOPO-TA cloning kit (Invitrogen) followed by sequencing using the Sanger method via a sequencer, to identify the sequences of individual aptamer candidates. The following five sequences were characterized to confirm that they are actual mTECs specific aptamer candidates (Table 1).

The five DNA aptamers (AraHH001, AraHH004, AraHH008, AraHH009 and AraHH022) were examined in a binding assay, using flow cytometry (Fig. 3). Among the five aptamer candidates, AraHH001 was found to bind to mTECs with high affinity. The binding affinity for the other four aptamers was low. Sequencing revealed that the full length aptamer was generally flanked by both primer sequences. We also found that only some of the nucleotides were responsible for binding. We first truncated both the full length sequences as well as the insert sequences of the selected aptamers that bind to mTECs. In these cases, the binding ability of the insert sequences was about equal to or better than the full length sequence. Therefore, we used the insert sequences of the selected aptamers in all of the experiments in this report. The use of a short, low molecular weight aptamer has many advantages in the design of drugs, and targeting devices for drug delivery for different therapeutic purposes. Aptamers with short sequences are also cost effective. The flow cytometry binding assay demonstrated that a high affinity DNA aptamer (Fig. 3) was successfully developed from our target primary cultured tumor endothelial cells using the cell-based SELEX method.

The mean fluorescence intensity measurement of various concentrations of the selected AraHH001aptamer, during binding assays with mTECs by flow cytometry, was used to determine the dissociation constant, Kd. The experiments were repeated three times. The AraHH001 binding affinity, (Kd, = was 43.8±13.7 nM). We also attempted to measure the Kd for AraHH001 on skin-ECs. (Fig. 4), but, fortunately, it did not bind to skin-ECs.

### Binding Assay of the Selected FITC-labeled AraHH001 Aptamer Against a Series of Non- targeted Cells and Cell Lines by Flow Cytometry

Although we performed two rounds of negative selection against the normal skin-ECs and OS-RC-2 cell lines at the 11^th^ and 12^th^ cycles, respectively, along with positive selection, it was very important to verify that the cell-SELEX produced aptamer actually binds specifically to the target mTECs. A binding assay with the AraHH001 DNA aptamer was performed to evaluate its binding capacity to various cells, including skin-ECs, OS-RC-2, RFP-SM, HUVEC, HMVEC, mOS-RC-EC, and hTECs by flow cytometry (Fig. 5). The AraHH001 mTECs selected DNA aptamer did not bind to skin-ECs, OS-RC-2 or RFP-SM cell lines (Fig. 5 A–C), and also failed to bind HUVEC and HMVEC (Fig. 5 D–E). This aptamer clearly bound cultured mOS-RC-ECs from another origin (Fig. 5 F). The high affinity binding of AraHH001 to hTECs (Fig. 5 G) has expanded the possibilities of its application to actual patients as well.

In order to determine the effects of trypsin on mTECs, and also to investigate the location of the binding target of the aptamer AraHH001, we pretreated with mTECs with trypsin, and then performed a binding assay by flow cytometry (Fig. 6). The binding of AraHH001 was very low. Therefore, the target site of the aptamer AraHH001 might be surface membrane proteins. These results also served to clarify the advantage of using RepCell dishes to maintain the natural state of the surface proteins of mTECs (Fig. 6), so that the aptamer could bind efficiently.

### Investigation of the Biological, and, or Functional Activities of AraHH001

After the successful development of the DNA aptamer, it was very important to assess its functions, including its anti-angiogenesis activities, on mTECs. The effects of AraHH001 on the angiogenic properties of mTECs were investigated by an *in vitro* tube formation assay (Fig. 7). The ability to form tubes and/or capillary-like structures was impaired by the treatment of mTECs with the aptamer AraHH001 in the matrigel. The same concentration of zero cycle libraries failed to interrupt tube formation by mTECs (Fig. 7 A). The tube lengths in the matrigel for each sample were quantitatively analyzed (Fig. 7 B). The inhibition of tube formation in the aptamer-treated sample was statistically significant, in comparison to the control group.

We investigated whether AraHH001 was assimilated by mTECs via CLSM, incubating FITC-tagged AraHH001 with mTECs. We observed the potential internalization of AraHH001 in mTECs by CLSM. To determine the exact location of the FITC-tagged AraHH001 after internalization, we used LysoTracker Red. The overlapping of FITC-tagged AraHH001 and LysoTracker Red confirmed that the DNA aptamer AraHH001 was internalized in acidic compartments (Fig. 8).

## Discussion

The formation of new blood vessels via tumor angiogenesis acts as a promoter for tumor cells to metastasize to distant organs, and, thus represents an opportunity to combat cancer. To address this problem, we developed a very simple technique, called cell-based SELEX, a modification of the usual SELEX technique using live cells. The technique involved the use of primary cultured cells, rather than establish cell lines as a target to isolate specific molecular probes or molecular ligands. Our flow cytometry binding assays of the isolated aptamer AraHH001 revealed very high affinity for two different origins of cultured mTECs, (Figs. 3, 5 F) and, high affinity binding to hTECs (Fig. 5 G).

During the selection process, we included some modifications that enhanced the success of the project. For adherent cells, detachment from the culture dish surface is a very important step in the selection process. Our binding assay results (Fig. 6), comparing the trypsin-pretreated target cells and the RepCell-collected target cells, revealed that this problem was resolved by maintaining the target cells under conditions that were as close to natural as possible. We also included a negative selection after 10 cycles, when we were confident that sufficiently enriched mTECs binding pools had been produced (Fig. 2). For the counter selection, we successfully used the normal skin-ECs and OS-RC-2 cell lines at the 11^th^ and 12^th^ cycles respectively, with positive selection. Our binding assay of the aptamer AraHH001 against a wide variety of cells and cell lines (Fig. 5) suggested that the negative selection doesn’t have to start from the first cycle with positive selection. As a negative control, we always used a fluorescently tagged zero cycle for unselected ssDNA pools that were amplified by a thermal cycler. Selection is a continuous and repetitive process, and results in the production of high affinity targets from random oligonucleotide pools. In our case, the use of an increased amount of randomly selected ssDNA pools (about 400 pmol) started after 5 selection cycles, which resulted in better enrichment of the random ssDNA pools in the selection [25]. As a result, after 12 cycles of selection, we were able to isolate the DNA aptamer AraHH001, which showed high affinity binding that was superior to those of the other four evolved sequences. This aptamer AraHH001 showed strong and specific binding toward the two different origins of mTECs, and also toward renal hTECs isolated from a renal carcinoma patient, and thus promises to be therapeutically useful for the identification of biomarkers. For a more in-depth understanding of the expression of cell surface proteins on tumor endothelial cells, proper and specific ligands are needed. Therefore, the successful internalization of this aptamer in an acidic compartment of mTECs has proven its promising application for developing ligand-based drug delivery. We plan to continue our investigations of the chemistry of AraHH001 and to identify the target biomarkers. An important concern is the heterogeneity of tumor endothelial cells, however, our isolated tumor endothelial cells have been tested, and reports have confirmed that their quality is maintained during long term culturing [4]–[6], [41]–[43]. Our isolated DNA aptamer AraHH001 has been shown to bind to cultured tumor endothelial cells of different origins (Figs. 3, 5 F–G).

The advantages of the whole live cell-SELEX system, include, no need for a detailed study of the target before the start of the selection; aptamers bind to the target in their original conformation; flow cytometry assays can be used for both the selection and, affinity analysis, and trypsin treatment of live cells with the target aptamer may confirm the nature of the protein target and whether it is a membrane protein (Fig. 6) [10]–[15], [27]–[29]. The present findings have shown that the selected DNA aptamer could be used as a molecular probe to differentiate normal skin-ECs, from mTECs. This aptamer also binds to hTECs under the same conditions, and the similar affinity will be helpful for understanding the molecular basis of the interaction. It is noteworthy that the selected aptamer was capable of inhibiting angiogenesis, and to inhibit tube formation (Fig. 7), indicating that is has promise for use in therapy. Additionally, our aptamer was assimilated within acidic compartments (Fig. 8), as evidenced by CLSM [44]–[46], thus opening an exciting therapeutic window for targeting the tumor vasculature, for the treatment of angiogenesis either by the aptamer itself or by developing a new drug delivery system.
